# Ingestion of Carbohydrate Solutions and Mouth Rinse on Mood and Perceptual Responses during Exercise in Triathletes

**DOI:** 10.3390/gels8010050

**Published:** 2022-01-09

**Authors:** María Martínez-Olcina, Laura Miralles-Amorós, Nuria Asencio-Mas, Manuel Vicente-Martínez, Rodrigo Yáñez-Sepúlveda, Alejandro Martínez-Rodríguez

**Affiliations:** 1 Department of Analytical Chemistry, Nutrition and Food Science, Faculty of Sciences, University of Alicante, 03690 Alicante, Spain; maria.martinezolcina@ua.es (M.M.-O.); laura.miralles@ua.es (L.M.-A.); niam1@gcloud.ua.es (N.A.-M.); 2 Faculty of Health Science, Miguel de Cervantes European University, 47012 Valladolid, Spain; mvmartinez11006@alumnos.uemc.es; 3 Escuela de Educación, Pedagogía en Educación Física, Universidad Viña del Mar, Viña del Mar 7055, Chile; rodrigo.yanez@uvm.cl; 4 Alicante Institute for Health and Biomedical Research (ISABIAL Foundation), 03010 Alicante, Spain

**Keywords:** gels, carbohydrates, mouth rinse, swimmers, sport, performance

## Abstract

Triathlons are endurance events that include swimming, running, and cycling. Triathletes need to eat optimally during training and competitions to maximize their potential for success. The presence of carbohydrates in the mouth could activate regions in the brain to enhance athletic performance in exercise. Methods: This study examined the effects of glucose and mouthwash in ten male triathletes (age: 26.0 ± 8.7 years, height: 173.6 ± 10.4 cm, BMI 22.0 ± 1.7 kg/m^2^). The four oral test solutions included (A) Rinse with placebo, (B) Water + gel with placebo, (C) Rinse with 15% CH concentration, and (D) Water + gel with 15% CH concentration (25 g gel in 165 mL water). The Rate of Perceived Exertion (RPE), Sensation Scale (FS), Felt Arousal Scale (FAS), Profile of Mood States (POMS), blood glucose, sprints, and dietary habits were assessed in each subject. All preceded ingestion of the oral-based test solution during workouts. Results: RPE showed no significant differences for subjective perceptions. The same was observed for FS and sprints. FAS scores increased over time (*p* = 0.039) in all groups. POMS score increased significantly in group D (*p* = 0.041). There was no effect of time on plasma glucose levels (*p* = 0.737). As for correlations, positive correlations were observed between sprint and FAS variables (*p* = 0.011). Conclusions: It appears that CH intake correlates positively with mood, but in all other variables, there are no differences depending on the product.

## 1. Introduction

The number of different endurance sporting events is increasing annually [1]. Triathlons are endurance events that involve swimming, running, and cycling. They vary in distance from sprint races that can take as little as 1 h to complete to Ironman-length events that consist of a 2.4-mile swim, a 112-mile bike ride, and a 26.2-mile run [2,3]. Most competitions include participants with a wide range of fitness goals and individual goals: some triathletes want to compete, and others simply want to complete [4].

Triathletes need to optimally fuel themselves during training and competitions to maximize their potential for success. Athletes practicing this sport, especially the swimming discipline, require adequate power, speed, and endurance to achieve their goal [5]. In this regard, much attention is paid to the scientific study of various aspects of nutrition as a factor of energy synthesis in muscle tissues during prolonged physical activity [1]. Proper execution of a nutritional plan that provides optimal fuel can make the difference between a personal best and premature fatigue in the race, modifying power, energy recovery, or affecting the occurrence of gastrointestinal discomfort [4]. 

Swimming is a sport that demands specific nutritional requirements depending on the individual [6] where carbohydrates (CH) are the key element for sports performance [7]. CHs are the main source of energy for endurance athletes due to their importance as fuel for muscle and central nervous system (CNS) function during moderate to high-intensity endurance exercise [8].

The ergogenic effects of exogenous CH consumption during exercise are related to the preservation of skeletal muscle glycogen, prevention of hepatic glycogen depletion, and subsequent occurrence of hypoglycemia and/or facilitation of high rates of CH oxidation [9]. CNS effects of running with low CH availability include alterations in pacing, motor skills, concentration, and increased perception of fatigue [7,10].

Ingestion of CH via sports drinks, gels, or sports foods during prolonged training sessions is beneficial for maintaining energy availability. However, practical considerations unique to triathlon competition include the lack of opportunity to refuel during swimming [5]. The rate of oxidation assuming adequate fluid intake has not been affected by the manner in which CHs are consumed (sports drinks, gels, or solid food) [4,11,12].

However, little is known about whether this performance benefit differs between different forms of CH administration. Sareban et al. [13] conducted research examining the impact of CH ingestion from gel or liquid beverage on measures of performance and gastrointestinal comfort. Both intakes contained the same amount of CH, a 27 g CH gel (mal-todextrin, fructose) per serving with a glucose/fructose ratio of 2:1 (Power-Bar, Nestle, Vevey, Switzerland) was used, 3 per hour were consumed. The liquid beverage used was 54 g maltodextrin (Lamperts Maltodextrin, Berco, Kieve, Germany) and 27 g fructose (Fruktosum, Fagron, Barsbüttel, Germany) in 1 L of bottled mineral water. None of the supplements contained caffeine. Results from the study suggested that gel administration did not alter performance, but it appeared to be associated with reduced gastrointestinal tolerance [13]. This is consistent with the fact that ergogenic aids in liquid form are often preferred by athletes, decreasing competition anxiety due to faster gastric emptying of liquids compared to solid foods [10]. 

Numerous investigations recommend those athletes who struggle with feeding CH due to gastrointestinal intolerance consider a CH rinse in place of food [14,15,16]. Frequent mouth rinsing with CH solution every 5 to 10 min with 10 s contact between the oral cavity and a CH source appears to produce the most reliable performance benefit thought to occur due to neural effects on decreasing fatigue [4,17]. However, the systematic review by de Ataide e Silva et al. [16] showed that there is a large variation in mouth rinse protocols: the duration of the mouth rinse varies between 5 and 10 s, the number of mouth rinse repetitions during the performance test (4 to 12 times); and the type of solution (maltodextrin, lemon juice, glucose, artificial sweeteners, and saccharin). Different studies have suggested that the presence of CH in the mouth may activate regions in the brain to enhance athletic performance in exercise, although admittedly, this evidence is limited [17,18].

Gastrointestinal symptoms that may appear from exercise can negatively affect the enjoyment and results of running [19]. Therefore, it is important to take into account the effect of CH supplementation on sports performance by observing the perceptual responses regarding exercise intensity, mood, level of activation/excitement, the feelings perceived by the subject, and the affectivity of the subject, i.e., whether he/she feels pleasure or displeasure during physical exercise [20].

Samełko et al. [21] showed that depression and positive emotional state were predictive of outcome in high and mid-range competitions. In turn, Monteiro et al. [22] found that the most important reason for attrition from the sport was dissatisfaction.

The measurement of these parameters and the balance between training-induced fatigue and recovery can provide valuable information to help revise training plans [23]. In addition, it should be used by sport psychologists, physical trainers, and coaches to promote the peak performance of these athletes and decrease attrition [24].

The hypothesis of this research is that the ingestion of gels and mouthwash with CH will decrease the perception of exertion during training as well as the improvement of specific performance in swimming. 

In this context, the objective of the intervention was to examine the influence of CH gels and mouth rinses on perceived exertion, affect, activation, mood, and swimming performance in triathletes.

## 2. Results and Discussion

**Figure 1:**
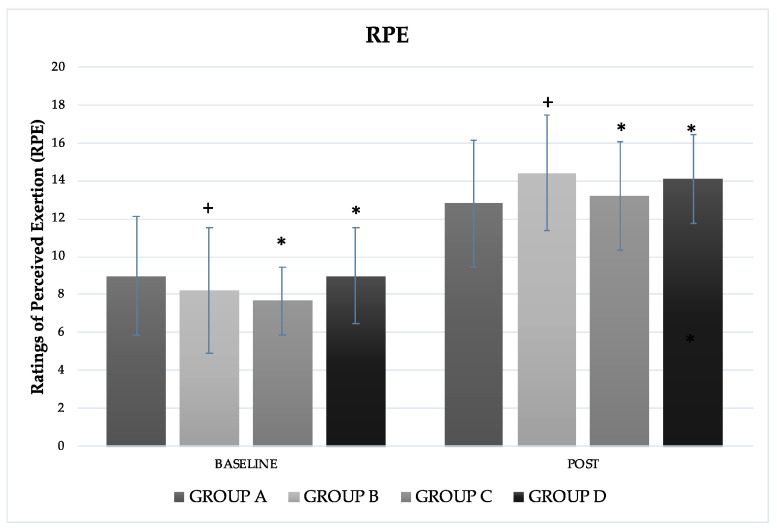
Ratings of Perceived Exertion (RPE) measured before and after the intervention. + = Significant differences *p* < 0.001. * = Significant differences *p* < 0.005.

Perceived Exertion (RPE), Figure 1, increased non-significantly (*p* = 0.053); from 9 ± 3.12 to 12.8 ± 3.35 in group A, while it increased significantly in group B (*p* < 0.001) from 8.22 ± 3.31 to 14.4 ± 3.05, in group C (*p* = 0.001) from 7.67 ± 1.8 to 13.2 ± 2.86, and in group D (*p* = 0.003) from 9 ± 2.5 to 14.10 ± 2.32. There were no differences between groups. The RPE is a scale to make a subjective evaluation of the intensity, that is to say, how you evaluate the repercussion of this on your organism. It is a description of the set of sensations that are produced, which are based on peripheral physiological, cardiorespiratory, and metabolic signals: tension in muscles and joints, state of the energy systems, perceived concentration of lactate, etc. [25]. The training itself, performed in all 4 cases under the same conditions (same load, intensity, and duration) modifies this perception, and it seems that the intake of different solutions and rinsing is not sufficient to cause differences between subjective perceptions. Previous research has shown significant improvements in the intake of placebo rinse or CH rinse [26,27,28]; however, the results obtained do not follow the same line.

**Figure 2:**
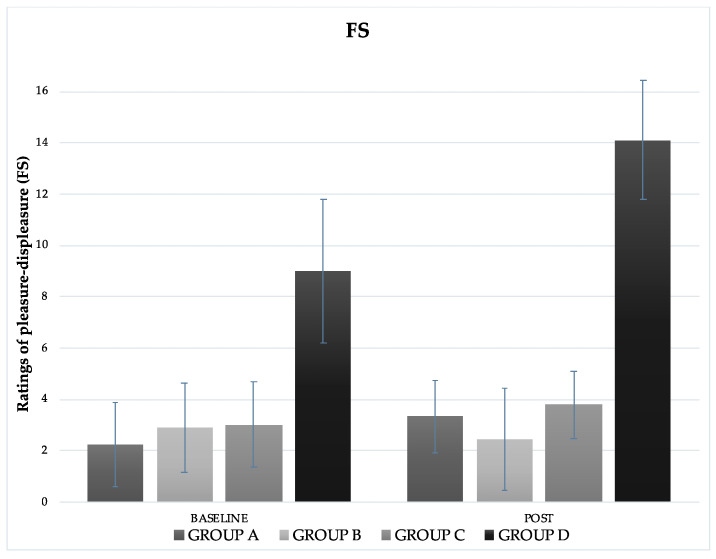
Mean ± SD of pleasure–displeasure ratings (Feeling Scale, FS) measured before and after the intervention.

FS ratings (Figure 2) decreased over time in group B (from 2.89 ± 1.76 to 2.44 ± 2.46) and increased in group A (from 2.22 ± 1.64 to 3.33 ± 1.41), group C (from 3 ± 1.66 to 3.78 ± 1.3), and group D (from 3 ± 1.22 to 2.78 ± 2.33). In none of the cases were the differences significant (*p* = 0.445), neither was there any time x treatment interaction (*p* = 0.446). 

The Feeling Scale (FS) was used to assess affective valence as it has been shown to correlate with other measures of core affect and several key concepts related to sport and exercise [29]. Considering the purported impact of CH mouthwash on areas of the brain associated with reward and pleasure, there is little research examining perceptual responses and mood during exercise [30]. Rollo et al. [31] reported that mouth rinse with CH significantly increased FS ratings (higher pleasant feelings) immediately before the 30-min run compared to rinsing with a placebo solution. However, this variation was not maintained once exercise began [31]; this finding is contrary to that of the present study in which no differences were observed at any stage of the trial (before and after the exercise intervention) between the different solutions and mouthwash.

**Figure 3:**
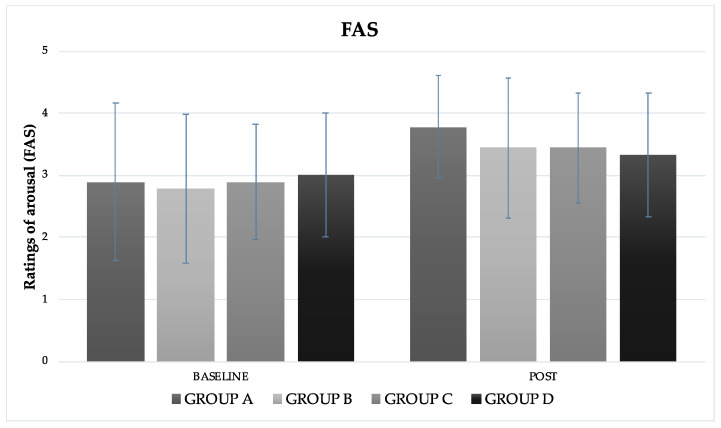
Felt Arousal Scale (FAS) responses (mean ± SD) before and after intervention.

FAS scores (Figure 3) increased over time (*p* = 0.039); values increased at each time point in all groups; group A (from 2.89 ± 1.27 to 3.78 ± 0.833), group B (from 2.78 ± 1.2 to 3.44 ± 1.13), group C (from 2.89 ± 0.928 to 3.44 ± 0.882), and group D (from 3.00 ± 1.00 to 3.33 ± 1.00). However, there were no interactions between treatment × time (*p* = 0.919).

Previous research [32] has found that FAS scores increase over time in athletes ingesting CH and decrease during placebo intervention. This does not coincide with the results obtained, since both the groups with rinse or placebo intake; A and B, respectively, and group C (rinse with CH) have presented improvements in the total score and therefore greater sensation of activation. 

The Profile of Mood States (POMS) is a common mood assessment test, which provides composite mood scores as well as several subscale scores [33]. In the present investigation, the overall composite POMS score (Table 1) increased in all groups after exercise. After performing the post hoc analysis, a significant increase was observed in group D (*p* = 0.041). As for the “tension” subscale, differences were observed over time but not between treatments. The post hoc analysis shows a significant increase in group C (*p* = 0.039). For the subscales “anger” and “depression”, there was no significant change neither over time nor between groups. For the “fatigue” subscale, significant increases were observed between baseline and after training in groups B (*p* = 0.020) and C (*p* = 0.016). For the subscale “vigor”, there was no effect neither in time nor as a function of the intervention. As for the “friendship” scale, the post hoc analysis showed a significant decrease in group D (*p* = 0.028).

The intake of CH gel (group D) is the only intervention that triggered significant responses in the total score of the questionnaire. Therefore, it seems that the intake of CH during physical exercise improves mood. It has previously been proposed [34] that CH intake before and during exercise helps to maintain optimal functioning of the CNS and, as a result, improve perceptual responses. Numerous studies have found that CH ingestion reduced mood states such as tension and fatigue compared to ingestion of a placebo solution [35,36]; however, the results obtained do not confirm this.

**Figure 4:**
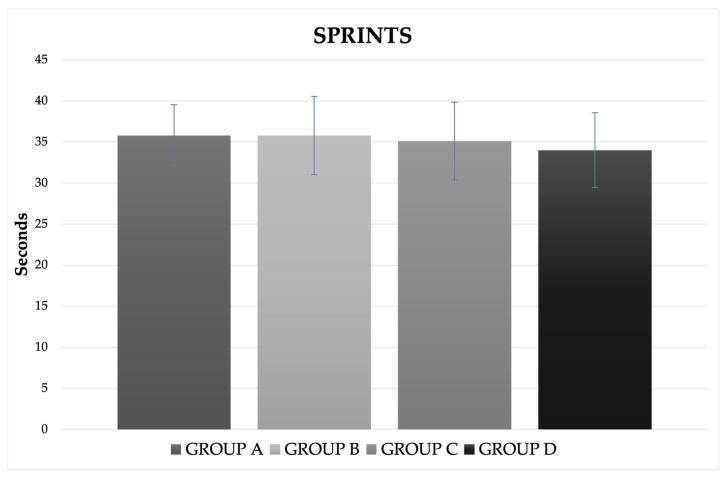
Time to perform 50 m (Mean ± SD) crawl-style swim of the different groups.

As shown in Table 2, there was no effect of time on plasma glucose levels (*p* = 0.737), nor were there differences depending on the intervention. As for sprints, there were slight differences between groups (Figure 4), but these were not significant either. Some investigations also did not observe a clear effect between CH and placebo in specific performance tests [26,37] or glucose [38], and some suggest that the use of a 5 s mouthwash with an isoenergetic amount of maltodextrin or glucose may not be beneficial for maximal sprint performance [39]. Methodological differences between the current study and these other studies, including the number of exercise tests, duration of resistance tests, mouth rinse dosage, resistance intensity, and training status of participants, may explain the disparity in performance and data.

As for the correlations (Table 3), positive correlations were observed between the sprint and FAS variables (*p* = 0.011); the greater the feeling of activation, the longer the sprint time; between sprint and the POMS subscales tension (*p* = 0.014), depression (*p* = 0.003), fatigue (*p* = 0.002), and total score, the higher the scores, the slower the sprints. Regarding the FS questionnaire, negative correlations were observed with the FAS questionnaire (*p* < 0.001), the different subscales and the POMS total score; higher FS scores, and therefore higher feelings of being at ease/satisfaction, correlated negatively and significantly with lower scores of the subscales anger (*p* < 0.001), depression (*p* = 0.006), fatigue (*p* = 0.003), and total POMS (*p* < 0.001), while it is positively correlated with the vigor subscale (*p* = 0.008). Finally, the RPE variable is positively related to the subscales anger (*p* = 0.004), depression (*p* = 0.021), fatigue (*p* < 0.001), friendship (*p* = 0.044), and total (*p* = 0.003); it seems that the perception of exertion is significantly related to “negative” feelings such as depression and fatigue. It is also negatively correlated with the FS questionnaire (*p* = 0.004); i.e., the higher the feeling of liking, satisfaction, the lower the values of exertion perception.

The present study has practical applicability and provides some insights; however, it cannot be extrapolated to the total triathlete population as no women were included in the research. Previously [40], it has been observed in female recreational runners that barnohydrate mouthwash did not improve 1 h running performance under low ovarian hormone conditions. Regarding the intake of gels, there is no research conducted in female athletes that analyzes the perceived effort, affect, activation and mood, but there is research that studies the muscle damage [41] of different CH and CH + protein solutions. They observe that the intake of CH + protein has greater benefits and that there are no significantly different responses between men and women. When studying the female athlete population, it is of great importance to know the menstrual phase in which each athlete is and the effect this has on performance, perception, and mood.

## 3. Conclusions

In conclusion, it seems that the intake of different solutions and gels with carbohydrates improves the perception of activation in swimming. The mood is significantly improved only in the group that ingested gel with CH; therefore, the intake of CH during sports practice has a positive influence on the psychological state of the athletes. In addition, a greater sensation of taste or satisfaction is related to a lower perception of effort.

## 4. Materials and Methods

### 4.1. Participants 

Ten male triathletes (age: 26.0 ± 8.7 years, height: 173.6 ± 10.4 cm, Body Mass Index (BMI) 22.0 ± 1.7 kg/m^2^) participated on a voluntary basis. All of them were moderately intermediate triathletes, who performed 10 to 15 h of training per week, interspersed with competitive events. They worked with them exclusively in swimming training. All participants completed a sociodemographic evaluation questionnaire, medical history, supplementation, and dietary record. The inclusion criteria were to have had a minimum of 3 years of continuous sports practice, to train at least 10 h per week excluding sporting events, not to suffer from any chronic disease (cardiovascular, diabetes), and to be of normal weight (18.5–24.9 kg/cm^2^) based on the WHO BMI tables [42].

### 4.2. Design 

This study presents a prospective experimental design. It is a clinical randomized controlled study. The same athletes acted both as a control group (placebo solutions) and as an intervention group (carbohydrate solutions). All procedures were previously approved by the Ethics Committee of the University of Alicante (UA-2021-03-11). Due to human experimentation, the ethical principles of the Declaration of Helsinki, which respects the human rights of all participants, were followed. Informed consents were supplemented by all participants in written form before starting. 

All participants took part in four experimental trials, for which one month of intervention was required. A 7-day period was established between each of the interventions. All trials were conducted at the same time, 08.00 p.m., and under the same temperature conditions, 28 °C. Participants were asked to avoid alcohol and caffeine consumption and to record dietary intake for 2 days before the first trial and to replicate intake before the other three trials.

### 4.3. Oral-Based Test Solutions

**Figure 5:**
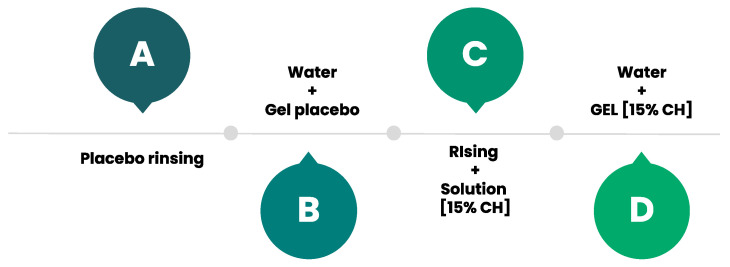
Intervention groups. A = Group A; B = Group B; C = Group C; D = Group D.

The intervention in the present study was based on CH delivery through mouthwashes and sports gels at different concentrations (Figure 5). The four oral-based test solutions included (A) Placebo rinse, (B) Water + placebo gel, (C) Rinse with 15% CH concentration, and (D) Water + gel with 15% CH concentration (25 g gel in 165 mL water). Participants were informed that they were to swallow the entire beaker when it was given to them. As for the mouthwash trials, they self-administered the mouthwash and were asked to keep it swishing in the mouth for 8 s and then expel it. Both solutions and rinse were administered at 12.5% of the completed training. The placebo solutions (Trials A and B) had the same taste and color and contained 0% CH and artificial sweeteners (stevia). The different solutions with CH (Trials B and C) also had the same color and flavor, the texture was similar; in the rinse, gelatin without CH were used to give it a less liquid, pastier, gel-like texture. Administration of the oral-based test solution and recipes were prepared according to previously established methods [30].

### 4.4. Outcome Variables

The validated versions of the Profile of Mood States (POMS), the Feeling Scale (FS), the Felt Arousal Scale (FAS), and the Rating of Perceived Exertion (RPE) were used to assess the perceived exertion, activation, and mood of triathletes in swimming training. All questionnaires were completed before and after each of the interventions, after 1 h of training.

#### 4.4.1. Rate of Perceived Exertion (RPE)

The RPE [43] is quantified by means of a scale ranging from 6 to 20 points ranging from “very light” to “very very hard”. It is used to evaluate the intensity of the exertion in a relative way according to each subject, since the same level and type of exertion can be perceived differently by each individual. It is a non-invasive, practical, and economical method that can be easily used by athletes to control the intensity of exercise in competition and training [44].

#### 4.4.2. Feeling Scale (FS)

When exercising, it is common to experience changes in mood. Some people find exercise pleasurable, while others find it unpleasant. In addition, the feeling can fluctuate over time. That is, one may feel good and bad several times during the same workout. The affective valence dimension was assessed by the Feeling Scale (FS) [45]. Participants rated their current feelings on an 11-point bipolar pleasure–displeasure scale, ranging from +5 (I feel very good) to −5 (I feel very bad). With intermediate points: I feel good (+3), I feel pretty good (+1), neutral (0), pretty bad (−1), and bad (−3) [46].

#### 4.4.3. Felt Arousal Scale (FAS)

The dimension of perceived arousal was assessed using the FAS [47], a 6-point scale ranging from 1 (low arousal) to 6 (high arousal) where athletes should circle the number that reflects their actual degree of arousal. During exercise, high arousal can be experienced as anxiety or anger or low arousal can be experienced as relaxation, boredom, or calmness. The FAS is strongly correlated with valid single-item measures used to assess arousal [48].

Both the FS and FAS have been used in several previous exercise studies conducted by various laboratories around the world and have demonstrated satisfactory convergent and discriminant validity [49].

#### 4.4.4. Profile of Mood States (POMS)

The POMS in its short version [50] is a goal checklist consisting of 30 items rated on a 5-point scale ranging from “not at all” = 0 to “very much” = 4; 6 factors are derived: anger (11 items), fatigue (6 items), vigor (5 items), friendship (6 items), tension (7 items), and depressed state, (9 items). Four of them are negative theoretical components (tension, anger, depression, and fatigue), and two are positive theoretical components (vigor and friendship). The total score is obtained through the sum of the factor scores.

#### 4.4.5. Blood Glucose

For this purpose, a glucometer of the brand “Free style” [51] and needles and lancets of the same were used. The blood testing procedure followed the usual standard process. This requires a blood sample, which is usually obtained by pricking the finger.

#### 4.4.6. Sprints

The swimmers underwent time trials swimming the full front crawl technique. All speed swimming performance tests were performed in each of the interventions, scored in seconds, and were determined by two expert timekeepers by stopwatch (Seiko S120-4030, Tokyo, Japan). For the 50 m arm pull front crawl tests, the swimmer was asked to cover the distance at maximum speed and with an individual start from out of the water to avoid the leg movement effect.

#### 4.4.7. Dietary Habits

By means of a dietary record administered and with the help of the easy diet program, both qualitative and quantitative assessments were made of the food they were eating at that time. As for the qualitative assessment, we can say that the average number of intakes was 5, they did not have any specific food habits for the pre-per-post training. In general, they should increase their vegetable intake; more than 50% have a very low intake of vegetables. The most problematic intake seemed to be breakfast; ultra-processed products (muffins, chocolate muesli, etc.) prevailed over more adequate options. Cooking quality seemed to be adequate; the main cooking methods were griddle, oven, or steam. Water intake should have been higher and there was generally no intake of supplementation; the few who did took “Recovery” for post-training recovery. Finally, as for the quality of the nutrients, most carbohydrates were slow-absorbing (bread and pasta), but there were also fast-absorbing sugars such as fruit and pastries. Proteins were mainly of high biological value (white meat), fish consumption was low, and cold cuts were abundant. The main source of fat was EVOO. As for fiber, it was not very high, since there were few vegetables, and the consuming HC were refined. 

### 4.5. Statistical Analysis 

Jamovi statistical software (Sydney, Australia) was used for statistical analysis. In addition to descriptive statistics (mean ± SD), a two-way repeated measures analysis of variance (ANOVA) was performed to examine (1) the different effects of the solutions, (2) time, and (3) group x time. Correlations between variables were examined using simple linear regression equations and reported as Pearson’s correlation coefficient (r). A small (weak) correlation was defined as ±0.10 to ±0.29, medium (moderate) correlation was defined as ±0.30 to ±0.49, and large (strong) was defined as ±0.50 to ±1.00 [52]. Data are presented as means ± SD. Statistical significance was set at *p* < 0.05.

## Figures and Tables

**Table 1:** Score of POMS scale before and after intervention.

	Group A				Group B				Group C				Group D									
	Baseline		Post		Baseline		Post		Baseline		Post		Baseline		Post		Effect Time			Effect Time × Group		
	Mean	SD	Mean	SD	Mean	SD	Mean	SD	Mean	SD	Mean	SD	Mean	SD	Mean	SD	F	*p*	η^2^*_p_*	F	*p*	η^2^*_p_*
TENSION	2.89	2.15	4.22	3.11	3.22	1.92	5	3.08	2.11	2.15	5.22	3.63	2.56	5.00	2.83	4.12	21.924	<0.001	0.407	0.706	0.555	0.062
CHOLERA	1.33	1.87	1.56	2.83	1.11	1.96	1.78	2.28	1.67	2.29	1.22	1.79	2	2.22	2	3.31	0.293	0.592	0.009	0.553	0.650	0.049
DEPRESSION	1.67	2.06	1.22	2.39	1.33	2.35	1.33	1.58	1.44	1.74	1.22	1.64	1.56	2.00	2.13	2.74	0.0556	0.815	0.002	0.648	0.590	0.057
FATIGUE	4.22	3.49	7.89	4.57	4.22	2.86	8.33	3.84	3.33	2.96	7.56	2.3	4.11	2.76	7.56	3.5	46.085	<0.001	0.590	0.104	0.957	0.010
VIGOR	11	2.65	13.3	2.69	12.3	3.24	12	2.55	12.7	3.04	13.3	2.74	12.1	2.71	11.9	2.93	1.22	0.277	0.037	1.240	0.310	0.104
FRIENDSHIP	13.9	3.18	12.7	2.45	14	2.87	11.8	3.56	13.3	3.67	12.1	5.33	13.7	3.57	10.4	3.97	18.2	<0.001	0.363	1.070	0.375	0.091
POMS TOTAL	85.2	9.22	88.9	9.53	83.6	9.91	92.7	9.22	82.6	11.0	89.8	9.11	84.4	9.85	94.4	11.4	24.823	<0.001	0.437	0.869	0.468	0.075

SD = Standard deviation; F = effect; η^2^*_p_* = partial eta square (effect size); *p* = *p* value; POMS = Profile of Mood States.

**Table 2:** Effect on plasma glucose before and after intervention.

	Group A				Group B				Group C				Group D									
	Baseline		Post		Baseline		Post		Baseline		Post		Baseline		Post		Effect Time			Effect Time × Group		
	Mean	SD	Mean	SD	Mean	SD	Mean	SD	Mean	SD	Mean	SD	Mean	SD	Mean	SD	F	*p*	η^2^*_p_*	F	*p*	η^2^*_p_*
GLUCOSE	95	18	97.8	12.2	99.1	12	86.8	5.91	90.2	9.85	93.2	7.68	91.3	8.25	93.9	18.7	0.115	0.737	0.004	1.636	0.201	0.133

SD = Standard deviation; F = effect; η^2^*_p_* = partial eta square (effect size); *p* = *p* value.

**Table 3:** Correlations between variables.

	GLUCOSE	SPRINT	FAS	FS	RPE	TENSION ANXIETY	ANGER HOSTILITY	DEPRESION DEJECTION	FATIGUE INERTIA	VIGOR ACTIVITY	FRIENDSHIP
GLUCOSE	—										
SPRINT	−0.259	—									
FAS	−0.186	0.420 *	—								
FS	−0.238	0.056	0.526 **	—							
RPE	0.172	0.258	0.058	−0.468 *	—						
TENSION–ANXIETY	−0.083	0.407 *	−0.031	−0.247	0.151	—					
ANGER–HOSTILITY	−0.034	0.310	−0.138	−0.626 **	0.465 *	0.500 *	—				
DEPRESSION	−0.044	0.474 *	−0.088	−0.451 *	0.382 *	0.379 *	0.876 **	—			
FATIGUE–INERTIA	0.175	0.490 *	0.069	−0.475 *	0.810 **	0.181	0.630 **	0.631 **	—		
VIGOR–ACTIVITY	−0.070	0.286	0.432 *	0.437 *	−0.256	0.085	0.046	0.095	−0.001	—	
FRIENDSHIP	0.094	0.037	0.051	−0.119	0.338 *	−0.445 *	0.158	0.287	0.491 *	0.105	—
TOTAL SCORE	−0.002	0.407 *	−0.182	−0.592 **	0.484 *	0.780 **	0.774 **	0.661 **	0.527 **	−0.259	−0.305

FAS = Felt Arousal Scale; FS = Feeling Scale; RPE = Ratings of Perceived Exertion; * = mean differences were significant at *p* < 0.005; ** = mean differences were significant at *p* < 0.001.

## Data Availability

The data presented in this study are available on request from the corresponding author. The data are not publicly available due to is personal health information.
